# Pediatric parosteal osteosarcoma of the distal radius causing extensive erosive mass effect of the adjacent ulna: a case report

**DOI:** 10.1186/s12891-023-07018-0

**Published:** 2023-12-06

**Authors:** Andrea Perloff, SeHoon Park, Robert Panganiban, John deVries

**Affiliations:** Orthopedic Surgery Department, Kirk Kerkorian School of Medicine at University of Nevada Las Vegas, Las Vegas, United States of America

**Keywords:** Parosteal Osteosarcoma, Pediatric, Distal Radius, Ulna, MDM2 gene amplification

## Abstract

**Introduction:**

Parosteal osteosarcomas are low-grade bony malignancies that are treated primarily with surgical resection and reconstruction. This report discusses a unique case of a pediatric patient who presented with a parosteal osteosarcoma of the distal radius causing extensive erosive mass effect and growth disturbance of the adjacent ulna. Likely due to their slow-growing nonaggressive nature, parosteal osteosarcomas have not been previously described to abut adjacent bony structures through direct contact. The patient presented in a significantly delayed manner due to social circumstances, inadvertently revealing this novel behavior. This report reviews this rare case and describes the current understanding of this tumor.

**Case presentation:**

The patient is a 13-year-old male who presented with a parosteal osteosarcoma of his distal radius. He presented with a palpable wrist mass and wrist stiffness. He presented in a delayed manner with advanced local disease due to social factors. Imaging revealed an osseous radial mass that abutted the ulna and likely stunted its growth. The patient ultimately underwent complex resection and allograft reconstruction of both his distal radius and ulna. Intraoperative pathology was confirmed to have negative tumor margins. Allograft reconstruction of the radius and ulna was performed utilizing patient-specific custom cutting guides. At the 6-month postoperative visit, the patient had no recurrence of the mass, minimal pain, and had almost regained range of motion of the extremities. Clinical radiographs at the 6-month postoperative visit demonstrated allograft incorporation.

**Conclusions:**

A previously unreported case of pediatric parosteal osteosarcoma of the distal radius with erosion of the adjacent ulna through direct contact is presented. The challenges in and the importance of arriving at a definitive diagnosis in a timely manner for the proper treatment of this malignancy are emphasized.

## Background

Parosteal osteosarcomas are low-grade bony malignancies that typically occur on the surface of metaphyseal long bones. They occur more commonly in women in the third decade of life. They carry a reported 16% risk of dedifferentiation and metastatic spread and are managed primarily with wide surgical resection and reconstruction [1]. Although other more aggressive bony malignancies have been shown to directly affect adjacent bony structures, parosteal osteosarcomas have not been described to behave in this way. Furthermore, it is even more rare for this tumor to cause an erosive mass effect on the adjacent bone causing a growth disturbance.

There are several diagnostic challenges present in identifying parosteal osteosarcomas. The histopathology of a parosteal osteosarcoma can appear benign. Both imaging and pathology can mimic other benign processes such as an osteochondroma [2]. Molecular testing such as MDM2 gene amplification is crucial in making the correct diagnosis [3, 4]. Furthermore, aberrant gene signaling is only detectable at the active margins of the tumor, making it essential to utilize meticulous biopsy techniques and obtain samples from multiple areas of the tumor [3].

This report presents an unusual case of a 13-year-old skeletally immature male with a parosteal osteosarcoma involving the distal radius with erosive mass effect of the adjacent ulna. Due to social factors, the patient presented in a delayed manner. Treatment was further delayed due to difficulties diagnosing the lesion. The patient ultimately underwent successful wide resection with negative margins and reconstruction with a strut allograft, plates and screws.

## Case presentation

**Fig. 1:**
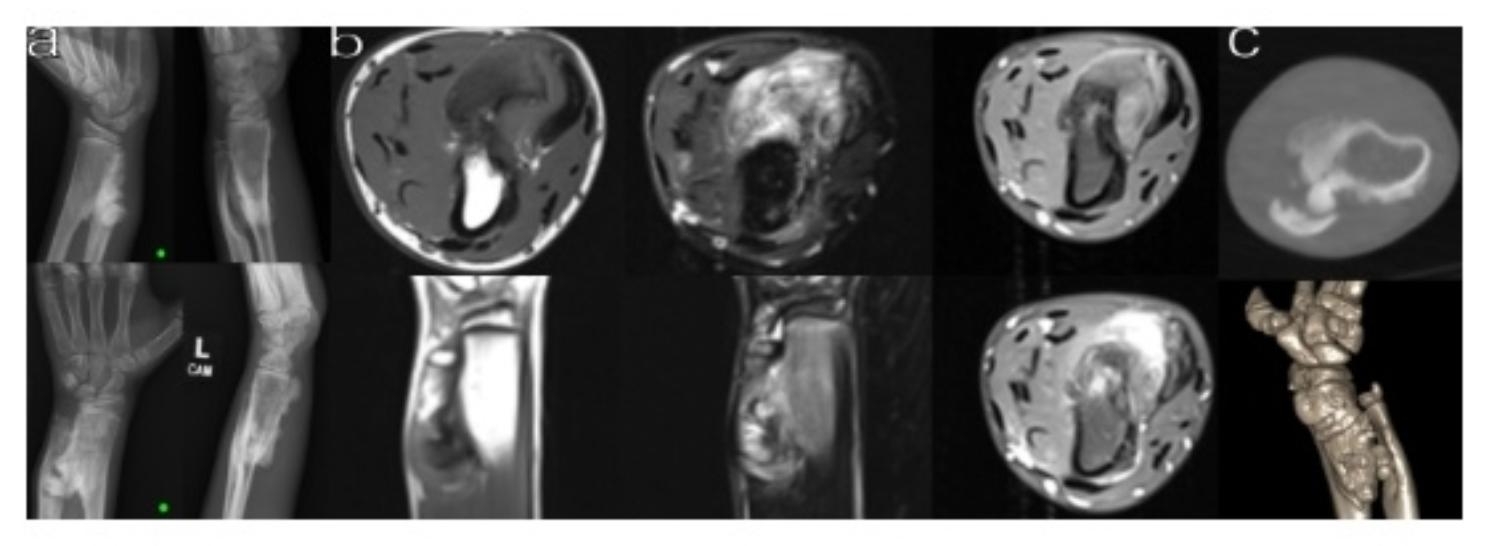
**a-c**: Preoperative imaging. **a** Radiographs of the left wrist demonstrating the parosteal osteosarcoma. The two top radiographs are the original images taken before the patient was lost to follow-up. The bottom set are one year later. **b** MR imaging left to right show the heterogeneous T2 hyperintense and T1 isointense to hypointense, avidly enhancing exophytic cortical medullary mass emanating from the ulnar aspect of the radial metadiaphysis extending locally into the adjacent ulna. **c** CT three dimensional reconstruction used for operative planning

The patient was a 13-year-old male, right hand dominant football player who was referred for a left wrist mass. He was brought in by his step-mother, who had noticed the mass and was concerned about the patient’s progressive decrease in wrist range of motion. Upon chart review, the patient had radiographs of the forearm documenting a left distal radius mass one year prior to his initial visit with an orthopedic oncologist. Further history revealed that after presenting the previous year, the patient was lost to follow-up and did not obtain further imaging or treatment during the interim. Physical exam revealed a large solid volar mass at the wrist without any sensorimotor deficits. The radial and ulnar pulses were both 2+. He had limited range of motion at the wrist with approximately 15 degrees of supination and 10 degrees of pronation. He lacked approximately 15 degrees of flexion and extension compared to the contralateral wrist. New radiographs demonstrated that the mass had grown approximately 3 mm in width, 5 mm in length, and increased in radiopacity compared to imaging one year prior (Fig. 1). MRI with IV contrast was obtained, which showed an approximately 3.5 × 3.2 × 6.3 cm heterogeneous corticomedullary mass arising from the ulnar aspect of the radial metadiaphysis that appeared hyperintense on T2 and hypointense on T1 sequences. The mass was found to extend locally to the adjacent ulna causing cortical thinning and deformation (Fig. 1).

Significant negative ulnar variance was noted on the affected wrist. It appeared as though the tumor abutment caused growth delay of the distal ulnar physis. The Hafner Method was utilized to determine the degree of negative ulnar variance. The difference between the most proximal point to the radial and ulnar metaphysis (PRPR) and the difference between the most distal point of the radial and ulnar metaphysis (DIDI) were measured to be approximately 1.35 and 1.6 cm on initial presentation one year prior. Similarly, more recent radiographs demonstrated approximately 1.15 to 1.68 cm of ulnar shortening.

Differential diagnosis at this time included osteochondroma, melorheostosis, high grade surface osteosarcoma, bizarre parosteal osteochondromatous proliferation, and low grade parosteal osteosarcoma. An open surgical biopsy targeting the ulnar aspect of the mass under fluoroscopic guidance was performed. Intraoperative frozen sections revealed lesional tissue with benign fibrous tissue and bone. Biopsied tissue was decalcified for permanent histology preparation. Therefore, molecular studies on the samples obtained were not able to be done. Permanent tissue histology demonstrated hyaline cartilage merging with bony trabeculae and associated bland fibrovascular and fatty tissues. The biopsy was ultimately deemed nondiagnostic.

**Fig. 2:**
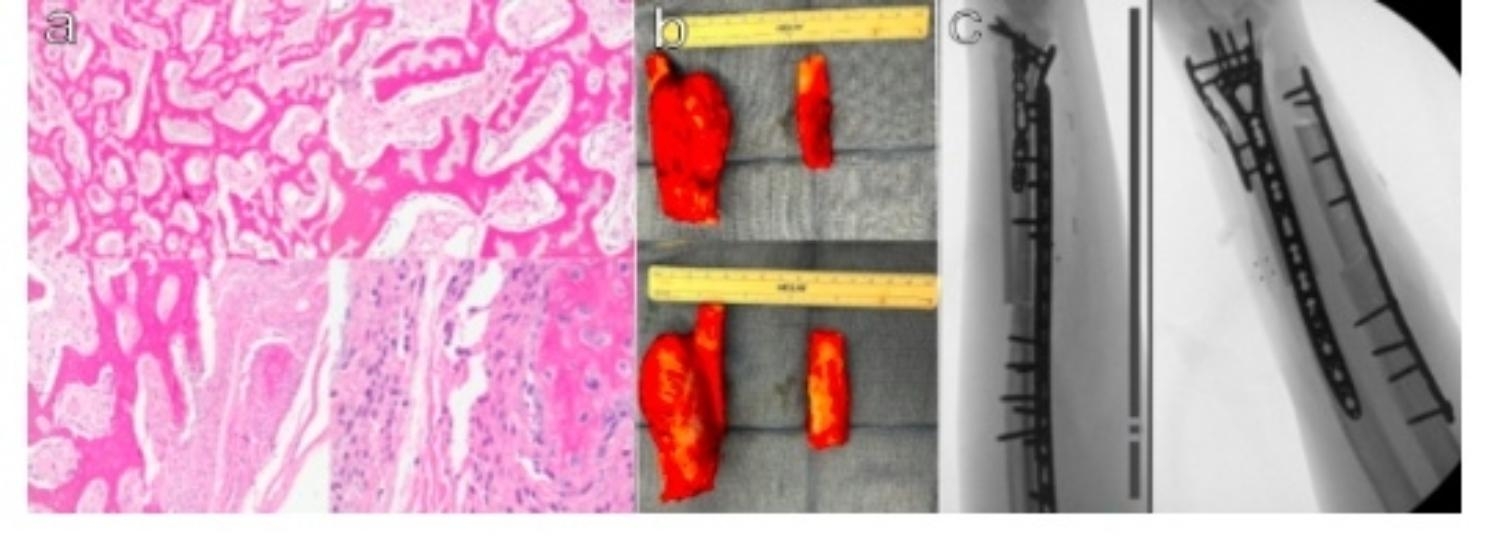
**a-c**: Operative pathology and imaging. **(a)** Histology of the distal radius showing a fibro-osseous lesion with hypocellular spindle-cell stroma containing trabeculae of bone. Spindle cells have minimal cytologic atypia and limited mitotic activity **(b)** Dorsal and volar photographs of the tumor resection of both the radius and ulna. **(c)** Post resection and reconstruction AP and lateral radiographs demonstrating plate and allograft placement

Due to the unique location, an inconclusive biopsy, and the tumor’s growth over the past year, there remained an ongoing suspicion of malignancy. Multiple discussions were had with the patient and family to perform a repeat biopsy of the mass and 3 months later a repeat open biopsy was performed following the initial procedure. Tissue was sent to an outside institution for consultation with molecular studies. The outside facility found the biopsy to be MDM2 positive on stain, and the patient was diagnosed with a low-grade parosteal osteosarcoma (Fig. 2).

Attempt was made to expediently schedule the patient for operative resection of the mass. However, the patient and family expressed the desire to complete his school football season despite inherent risks. The patient ultimately underwent wide resection and reconstruction of his left distal radius and ulna approximately 18 months after the mass was first found. Due to the tumor’s proximity to the wrist, priority was to obtain margin-negative wide resection and to maximize postoperative function with maintaining the radiocarpal and distal radioulnar joints while preserving the articular portion and preserve the DRUJ and radiocarpal joint. This was done using custom 3D printed patient-specific cutting guides were developed from preoperative CT scans (Fig. 1). The mass emanated from the ulnar side of the radius and was in direct contact with the ulna. The ulna was hypoplastic, with a notably thin cortex, which could potentially pose challenges for a hemicortical resection. Therefore, custom cutting guides were developed for both the radius and ulna so that precise total ulnar resection could be done if unable to complete a hemicortical resection. The radius cutting guide was designed to provide a geometric resection with close margins to preserve native bone stock, and provide native bone on the radial aspect, adding stability and increasing contact between host and graft for increased healing potential.

Intraoperatively, the distal radius was resected en bloc just proximal to the physis salvaging the distal articular segment as planned with the 3D generated cutting guide. The ulna was not resected en bloc with the radius so that frozen sections of the ulnar periosteum and cortical bone could be obtained separately to evaluate intraoperative margins and if full resection was indicated. Frozen section did show suspicion for invasion of tumor cells therefore the custom fitted ulna cutting guide was used for resection. The resected tumor measured 7.6 cm by 4.3 cm (Fig. 2). All neurovascular structures were identified and confirmed to be intact after resection. Matching distal radius and ulna fresh frozen allografts were selected preoperatively and cut to shape using 3D printed cutting guides that matched the preoperative planned resection and bone deficits from the resection. The allografts were fixed onto the proximal segments of the patient’s radius and ulna using plates and locking screws, respectively.

**Fig. 3:**
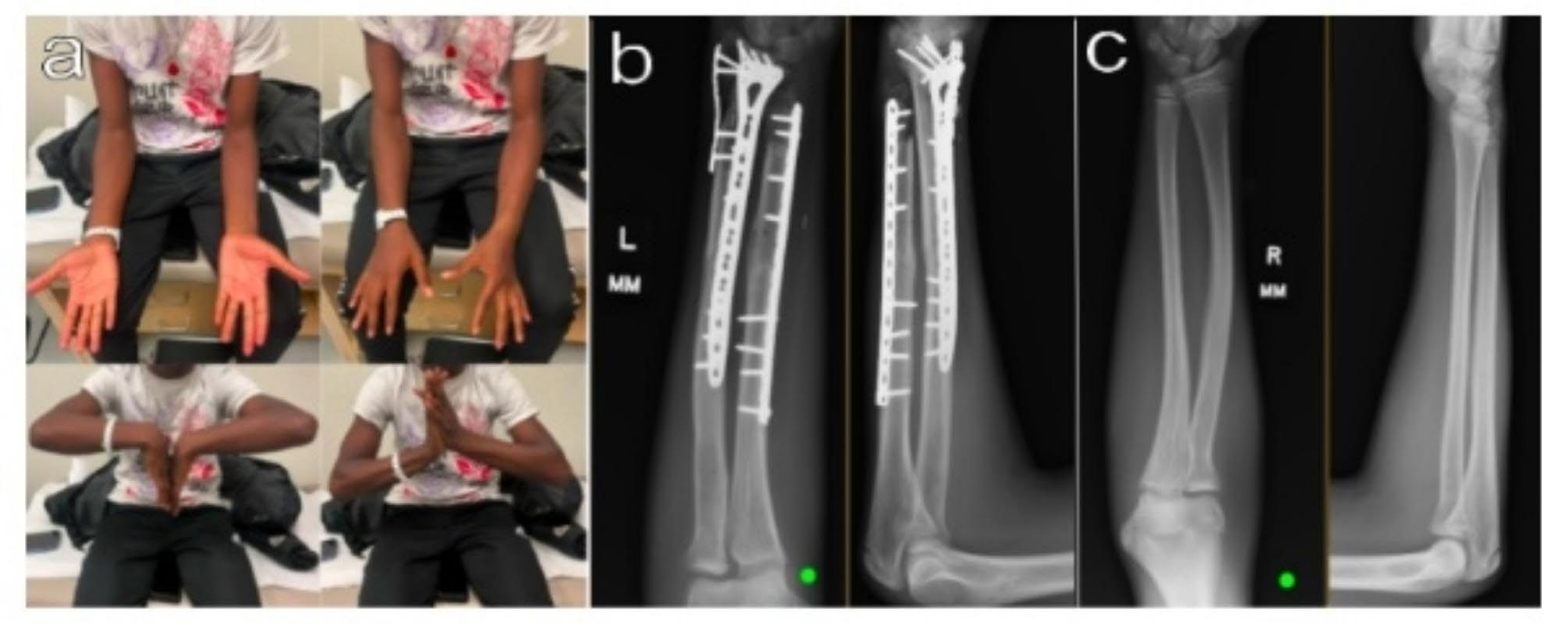
**a-c**: 6 months after surgical management. **a** Active range of motion with full supination of 90 degrees, 80 degrees of pronation, 83 degrees of flexion, and 75 degrees of extension. **b** Radiographs at six months show intact hardware and full incorporation of the radial allograft and continued incorporation of the ulnar allograft. **c** Radiographs of the contralateral forearm at the time of the patients 6 month postoperative appointment. These have been provided for reference to the amount of erosion the tumor caused to the left ulna

The patient had a normal neurovascular exam postoperatively. He was placed in a cast and made nonweight bearing to the extremity for three months. Final pathology sent to an outside institution (Mayo Clinic in Rochester, MN) concluded that there were negative margins for malignancy and confirmed the diagnosis of low-grade parosteal osteosarcoma of the distal radius. The Ulna did have reactive changes however it did not have MDM2 amplification which favored the radiographic changes being due to “extensive erosive mass effect” rather than tumor involvement. At his six-month postoperative appointment, the patient had almost regained full range of motion (Fig. 3). Radiographs show bony union at the allograft junction. At this six-month visit imaging of the contralateral forearm was taken showing neutral ulnar variance (Fig. 3). He has been overall satisfied with his progress and plans on playing football this fall.

## Discussion

Parosteal osteosarcomas are rare low-grade bony malignancies with a slight female preference found in the second to fourth decades of life [1]. Okuda et al. reported a female to male ratio of 1.28:1 in their report of 228 patients [1]. Parosteal osteosarcomas arise from the periosteum of metaphyseal long bones, most commonly in the distal femur, proximal tibia, and proximal humerus [1, 5]. However, there have been reports of parosteal osteosarcomas arising from various locations, such as the distal radius, talus, cranium, mandible, ribs, clavicle, and tarsal bones [6–8].

Although they were once thought to be a subtype of intramedullary osteosarcomas, the World Health Organization (WHO) now recognizes parosteal osteosarcomas as a distinct entity with its own unique clinical course and pathologic characteristics [9]. On presentation, patients most commonly complain of a painless growing mass and range of motion limitations in cases in which the tumor arises adjacent to a joint [10]. Radiographically, the tumor appears as a cortically based exophytic ossified mass without extension into the medullary canal [4]. They can often be mistaken for osteochondromas, which also appear as cortically based bony masses, but present with continuity of the medullary space between the mass and the native bone.

Histological and immunochemical analysis showed a fibro-osseous lesion with hypocellular spindle cell stroma containing trabeculae of bone. Spindle cells usually have minimal cytologic atypia and limited mitotic activity [1]. Okuda et al. delineated guidelines for identifying parosteal osteosarcomas that involve radiographic evidence of bony origin and histology showing well-formed osteoid, spindle-cell stroma and overall well-differentiated low-grade appearance. They also noted that in cases with medullary involvement, there must be less than 25% involvement of the cavity [1]. Molecular studies can aid in the diagnosis of this malignancy over its benign imitators. Yoshida et al. examined MDM2 and CDK4 gene amplifications to differentiate parosteal osteosarcoma from osteochondroma. The authors found that all of the parosteal osteosarcoma samples demonstrated positivity to CDK4 gene amplification, and the majority showed positivity to MDM2 gene amplification [3]. Duhmel et al. reported that MDM2 could be utilized to distinguish parosteal osteosarcoma from conventional osteosarcoma, as 85% of parosteal osteosarcomas in their sample were MDM2 amplification positive [4].

Parosteal osteosarcomas are well-differentiated malignant tumors with low local recurrence rate and metastatic potential. Treatment consists of wide resection without chemotherapeutic intervention. Ruengwanichayakun et al. demonstrated that the degree of dedifferentiation of these tumors had the largest inverse effect on long-term survival. They reported 96% 5-year survival in parosteal osteosarcomas compared to 65% in cases with dedifferentiation. Similarly, they reported the 10-year survival rates of the two groups to be 96% and 60%, respectively [11]. Their findings demonstrated that medullary involvement, age at presentation, and tumor size did not impact survival rates. However, achieving negative surgical resection margins is crucial for disease control [11]. Okada et al. similarly showed that with incomplete resection there was increase of local recurrence that dedifferentiation markedly increased the risk of metastasis [1].

In this case, there was a significant delay in treatment due to various social factors. The patient initially presented with advanced local disease and was lost to follow-up for an additional year. Surgical resection was further delayed following diagnosis due to the patient’s desire to participate in his high school sports season. Fortunately, despite the delayed presentation, the patient’s lesion did not worsen significantly on repeat plain radiographs. This reflects the slowly evolving natural history of this tumor.

Direct local abuttment of adjacent bone has been reported in aggressive bony malignancies and has been associated with a higher likelihood of local recurrence and poorer outcomes [12]. In this case, both preoperative imaging and intraoperative histology demonstrated tumor abuttment causing extensive erosion of the adjacent ulna. This phenomenon has not been characterized previously in parosteal osteosarcomas of the distal radius in skeletally immature patient. This may be due to the fact that these lesions more commonly arise in the distal femur, proximal tibia, or humerus, where direct bony abutment to an adjacent bone is far less likely. The delay in presentation also played a role in allowing the tumor to grow to a sufficient size to allow for adjacent bony abutment and subsequent growth disturbance. It may be that in previously reported cases, the tumor was resected prior to reaching a size to abut and efface an adjacent bone. Likely this phenomenon is only seen in later stages of the disease. Although this demonstrates the slow-growing nature of parosteal osteosarcomas, expedited treatment is recommended since a more aggressive resection and complex reconstruction may be required.

Utilizing the mean distal ulnar growth rate and negative ulnar variance measurements, an estimation of when the tumor began impeding the distal ulnar growth and approximate how long the tumor had been growing. The Hafner Method was utilized to calculate the ulnar variance for the patient. In short, this is a validated method of measuring negative ulnar variance in pediatric patients by measuring the difference between the most proximal point to the radial and ulnar metaphysis (PRPR) and the difference between the most distal point of the radial and ulnar metaphysis (DIDI). Even at initial presentation, the patient had approximately 1.35 to 1.6 cm of ulnar shortening. Assuming the mean growth rate of the distal ulna to be approximately 0.9 cm per year; the mean ulnar growth rate in skeletally immature males is reportedly 1.1 cm per year, with the distal ulna accounting for 85% of the growth [13]. Using these values, it was estimated that the tumor had been impeding the growth of the ulna for approximately 1.5 to 1.9 years even prior to initial presentation. Of note, this only accounts for when the tumor was of sufficient size to start impeding the growth of the distal ulna. Although an estimate, this does give insight into the age of the tumor and how slow growing it is.

Finally, the case also demonstrated the common diagnostic difficulties associated with this malignancy. Pathology can often have a benign appearance, making molecular studies crucial in making the correct diagnosis. Aberrant gene signaling can only be detected at the active margins of the tumor, and as in this case, these can be prone to sampling errors [3]. Adhering to careful biopsy techniques and sampling multiple areas of the tumor, making sure to include active borders of bony growth are paramount. Furthermore, communication with the pathology team regarding the differential diagnosis, clinical presentation, and molecular studies needed can shorten the time to diagnosis and treatment.

## Conclusion

Presented is a case of low-grade parosteal osteosarcoma in the distal radius in a skeletally immature individual. Described is a unique phenomenon direct contact between adjacent bones. To date, this has not been previously described in the literature for this malignancy. In addition, highlighted are the challenges encountered during the management of this case. Tissue diagnosis was obtained and limb salvage was possible in part due to the advances made in molecular testing for this malignancy. Although the diagnosis and treatment were delayed in this case for various reasons, the patient thus far has had a positive outcome, which provides insight into the unique natural history of this tumor.

## Data Availability

Written consent was obtained from the patient and parents for publication of the patient’s details. The data supporting this case report are from previously reported studies and datasets, which have been cited. The processed data are available upon request from the corresponding author.

## Abbreviations

3D: Three Dimensional
MDM2: Mouse double minute 2 homolog
MM: Millimeter
CM: Centimeter
MRI: Magnetic resonance imaging
IV: Intravenous
T2: Transverse relaxation time
T1: Longitudinal relaxation time
PRPR: Proximal point to the radial and ulnar metaphysis
DIDI: distal point of the radial and ulnar metaphysis
CT: Computerized tomography
AP: Anterior posterior
MN: Minnesota
WHO: World Health Organization
CDK4: Cyclin-dependent kinase 4
